# Arterial Microcalcifications in the Breast Mimicking Malignancy

**DOI:** 10.1155/2012/946317

**Published:** 2012-02-08

**Authors:** Katrin Janzen, Jan Janzen

**Affiliations:** ^1^Praxis für Gynäkologie und Geburtshilfe, Bollwerk 35, 3011 Berne, Switzerland; ^2^Praxis für Histopathologie, Postfach 350, 3000 Berne 22, Switzerland

## Abstract

Microcalcifications in the breast are highly suggestive of malignancy; they can occur in many pathological conditions. A 36-years-old nondiabetic woman came to the gynaecologist with a suspect palpable mass in the upper outer quadrant of the right breast. Histopathological examination confirmed a calcification of a small artery (diameter: 0.45 mm). Arterial calcifications can mimic a malignant lesion in the breast.

## 1. Introduction

Vascular calcifications as a form of crystallization are reflecting a complex biological mechanism. Calcifications can occur in many pathological conditions, for instance, in toxic injuries and in long-term treatment with corticosteroids. Furthermore, cell death in apoptosis and mitochondrial vesicles can be responsible for calcifications. Recent studies are focusing on the inhibition effect of Matrix-Gla protein [1].

## 2. Case Presentation

**Figure 1:**
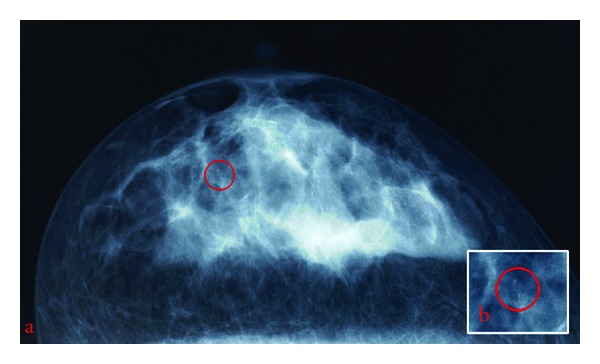
Radiologic aspects presenting small microcalcifications (a, b).

A 36-years-old nondiabetic woman came to the gynaecologist with a suspect palpable mass (1.5 cm in diameter) in the upper outer quadrant of the right breast. Radiologically, unclear groups of microcalcifications were detected in the mammogram (Figure 1). Three stereotactic 11-gauge vacuum-assisted breast biopsies were performed. Microscopically, fragmented breast biopsies with a total length of 6.0 cm were examined. Typical aspects of lobular hyperplasia characterised by enlarged and hypercellular lobules were found. In the adjacent stroma rare mononuclear inflammatory infiltrates and small fibrotic foci were localised. Furthermore a small muscular-type artery with circumferentially arranged smooth muscle cells in the tunica media was removed. Intimal and adventitial layers of the artery showed no pathological changes. However, in the tunica media amorphous deposits—stained blue and violet in hematoxylin-eosin—were observed. These microcalcifications had a diameter of 0.45 mm.

Our case showed typical aspects of calcifications in a muscular type artery, where calcifications develop alongside the internal elastic membrane [2]. Microcalcifications were removed by biopsies in toto. There were no signs of malignancy. Arterial calcifications frequently occur in elderly people (senile medial calcinosis, Moenckeberg's medial sclerosis) [3]. Diabetes mellitus and hyperlipidaemia have been reported with high prevalence in young and middle-aged woman with breast arterial calcifications [4].

It is well known that microcalcifications in the breast are highly suggestive of malignancy. Uncertain calcifications should indicate a further histopathological examination to confirm their biological behaviour [5–7]. So, benign microcalcifications associated with apocrine metaplasia in fibrocystic breast disease and after breastfeeding were diagnosed [8, 9].

## 3. Conclusion

Arterial calcifications are pathologic and can mimic a malignant lesion in the breast.
